# O.6.2-2 Building momentum: lessons learned in the promotion of physical activity in primary healthcare settings

**DOI:** 10.1093/eurpub/ckad133.272

**Published:** 2023-09-11

**Authors:** Kendra Chow, Katy Cooper, Rebecca Taylor, Kate Oldridge-Turner, Magdelena Wetzel, Kate Allen

**Affiliations:** World Cancer Research Fund International, UK; Independent Consultant, NCD Prevention; k.chow@wcrf.org; World Cancer Research Fund International, UK; World Cancer Research Fund International, UK; World Cancer Research Fund International, UK; World Cancer Research Fund International, UK

## Abstract

**Purpose:**

Physical activity plays a vital role in promoting and maintaining good physical and mental health, but too many individuals are not meeting recommendations. Policies that support physical activity prescription (PAP) within primary healthcare (PHC) settings are a key tenet of a comprehensive, systems-wide approach to increase physical activity and help prevent and manage many non-communicable diseases, including cancers. The fourth Building Momentum report sets out the emerging evidence for the benefits of promoting physical activity in PHC, and explains why designing and enacting such policies is good for individual health and economies. This is the first known report of its kind, offering a global policy perspective on developments and progress, as well as guidance on foundational policy processes and components.

**Description:**

Physical activity is a WCRF International policy priority, directly related to our cancer prevention recommendations and MOVING framework and policy database.

Research, writing and publication were managed by WCRF International. The current state of evidence and policies were evaluated through a literature review, and interviews conducted with subject-matter experts selected for their knowledge and experience.

The report was evaluated through two rounds of review, including by interviewees, physical activity experts, and individuals from the WCRF Network, Policy Advisory Group, and the WHO.

The report will be disseminated through an initial online launch event that will showcase the main findings, country reflections and guidance. Further dissemination will be through WCRF International’s health professional and policy networks, social media, and potentially conferences like HEPA.

**Conclusions:**

The fourth Building Momentum report provides guidance on how to establish successful policies for PAP in PHC. It addresses key issues and potential barriers to adoption, as well as identifying foundational policy processes, including: using the evidence, building shared policy objectives, the importance of local contexts and equity underpinning all policy elements. It also identifies components for designing effective policies, including: PHC professional training, health systems capacity, incentivizing structures, the importance of communication and collaboration, and creating supportive patient environments.

Engaging with these findings, as well as examples of national programmes, will enable governments and health systems to enact well-designed policy on physical activity promotion in PHC.

